# Anesthetic management of a pediatric patient with methylmalonic acidemia combined with hyperhomocysteinemia: A case report

**DOI:** 10.1002/ccr3.7924

**Published:** 2023-09-21

**Authors:** Yunting Pang, Fanqing Meng, Yaqiu Guo, Feng Zhou

**Affiliations:** ^1^ Department of Anesthesiology Jinan Maternal and Child Health Care Hospital Jinan China

**Keywords:** case report, general anesthesia, methylmalonic acidemia, strabismus

## Abstract

Methylmalonic acidemia (MMA) combined with hyperhomocysteinemia is an autosomal recessive genetic disease which can lead to metabolic acidosis, elevated lactate, and high blood ammonia level. This anesthetic management was mainly how to maintain the stable state of perioperative physiological metabolism of such patients.

## Graphical abstract

Patient of oncomitant esotropia with methylmalonic acidemia combined with hyperhomocysteinemia.

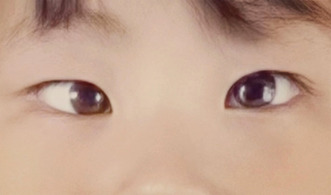

## 1 INTRODUCTION

Methylmalonic acidemia (MMA) is an autosomal recessive disorder with an estimated incidence of 1:500,000, and MMA with hyperhomocysteinemia of 1:60,000–36,000.^1^, ^2^ It is caused by congenitally abnormal cobalamin metabolism, where cobalamin plays an important role in methylmalonic acid and homocysteine metabolism. Notably, in cobalamin disorders, methylmalonic acid and homocysteine accumulate in the body, which can manifest as multiple organ damage.^3^ Patients typically present in the infantile period (<1 year) with failure to thrive, metabolic acidosis, thrombotic microangiopathy, and neurological symptoms such as lethargy, developmental delay, and muscular hypotonia.^4^ Furthermore, patients often present with additional megaloblastic anemia or neutropenia/pancytopenia. Metabolic acidosis, anemia, lactic acidemia, and hyperammonemia are key issues associated with anesthesia.^5^ Thus far, little is known about anesthetic management and the selection of anesthetic drugs. Here, we report the case of a child with MMA combined with hyperhomocysteinemia who underwent strabismus surgery under general anesthesia, and we discuss pertinent information related to anesthetic management.

## 2 CASE PRESENTATION

**FIGURE 1:**
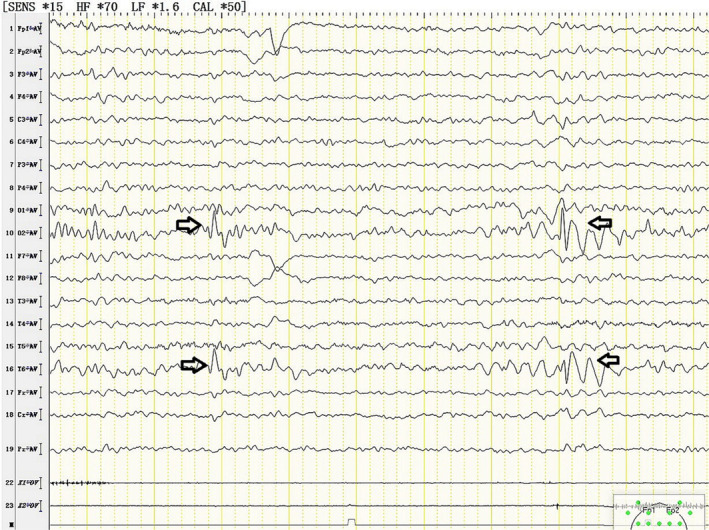
Abnormal electroencephalogram of the patient, as shown by black haircuts. In the conscious state, the right middle‐posterior temporal and occipital region had low and medium amplitude sharp waves and spike waves synchronously or asynchronously, which could spread to the adjacent leads. Discharge of right middle‐posterior temporal and occipital region.

**FIGURE 2:**
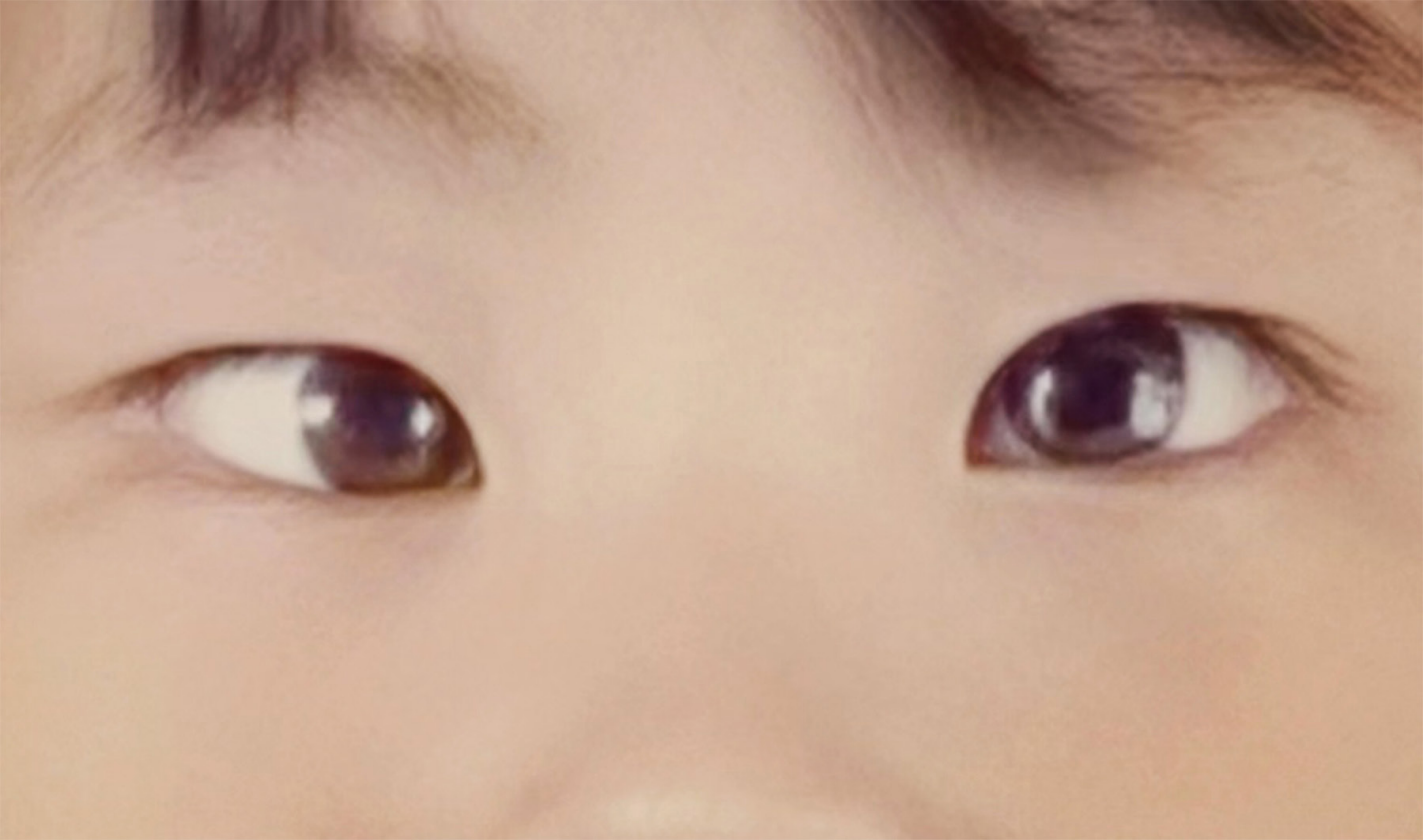
Clinical presentation of preoperative concomitant esotropia.

A 6‐year‐old Chinese girl was admitted to the hospital due to binocular strabismus. After birth, she was hospitalized for pulmonary hypertension and took 10 mg of bosentan tablets orally twice a day, for 4 weeks of treatment. She was diagnosed with MMA combined with hyperhomocysteinemia at 42 days after birth through a heel blood test, and genetic testing revealed heterozygous mutations at the c.599G>A and c.609G>A loci in the *MMACHC* gene. She received the following medication to maintain the stability of her condition: vitamin B_12_ 10 mg introduced intramuscularly, 1 time/3 days, in combination with the oral administration of betaine 0.3 g, 1 time/1 day, calcium folinate 5 mg, one time a day, and levocarnitine 1 g, 3 times a day. Her mother underwent surgery for esotropia 10 years ago, while her father had a history of vitiligo, for over 10 years, and her 15‐year‐old sister had a history of epilepsy, for over 10 years. Her sister and mother were carriers of the *MMA* gene. She weighed 25 kg, and her medical history included slight speech delay, intellectual disabilities, and behavioral disturbances. She did not have any dysmorphic facies, and her airway examination was normal. Her echocardiography showed no abnormalities, but the electroencephalogram did identify abnormalities (Figure 1). The laboratory results were normal, except for a plasma homocysteine level of 50.3 μmol/L (normal value, 0–15 μmol/L), a blood ammonia level of 55.2 μmol/L (normal value, 10–47 μmol/L), and a lactate level of 2.3 mmol/L (normal value, 0–4 mmol/L). Hence, correction of strabismus under general anesthesia was planned (Figure 2).

**FIGURE 3:**
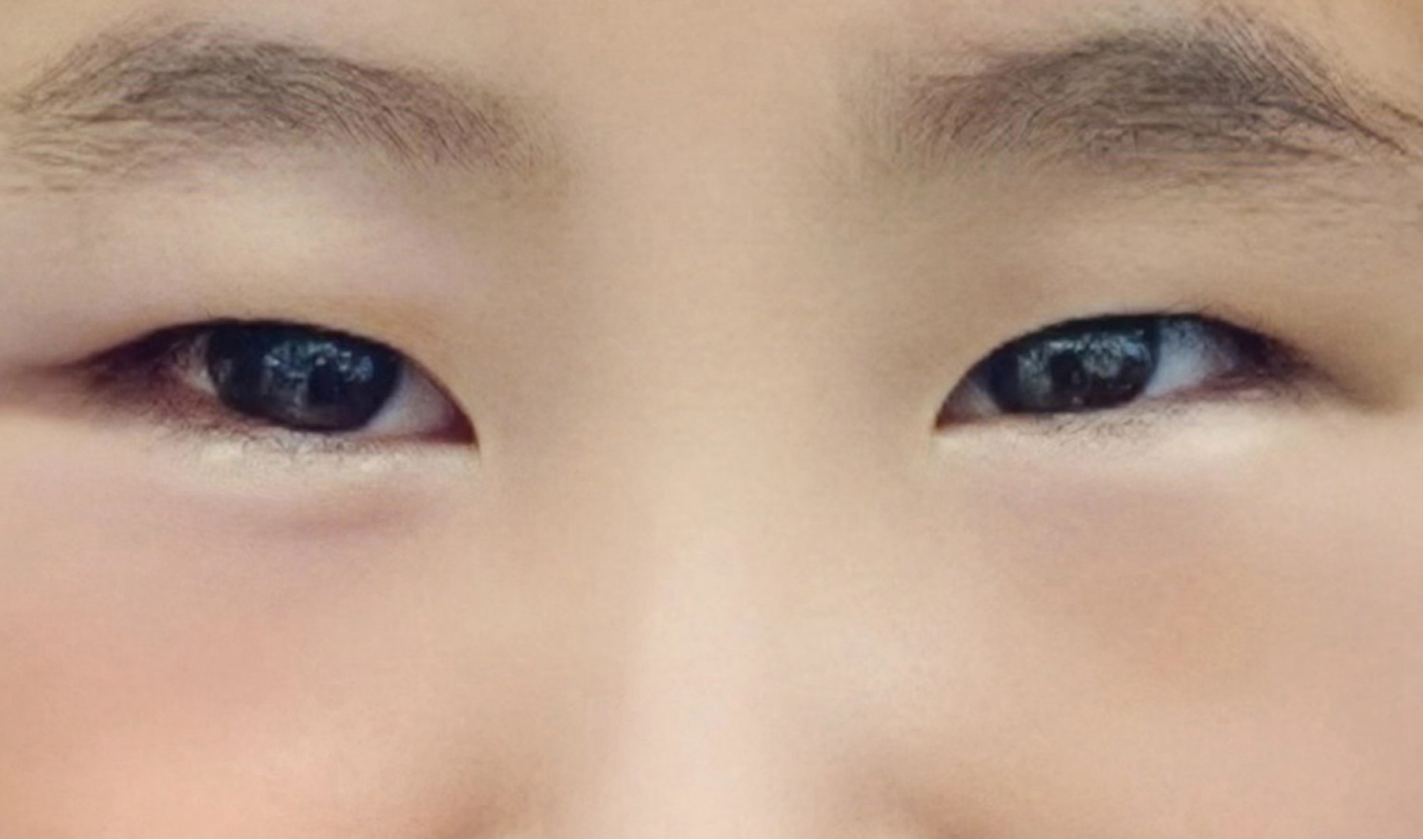
Clinical presentation of the eyes 6 months after the correction of strabismus under general anesthesia.

Upon arrival in the operating room, the patient was calm and was placed on a forced‐air warming blanket. In addition to cardiac rhythm, blood pressure and pulse oximetry, temperature monitoring was conducted. After securing IV access with sufentanil 5 μg IV, propofol 70 mg IV, penehyclidine 0.3 mg, and dexamethasone 3 mg was given, beside assisted ventilation. A 2.5 # flexible laryngeal mask was administered, and placement was confirmed. Propofol (10–15 mL/h) and remifentanil (0.1–0.2 μg/kg/min) were administered via continuous IV infusion. No muscle relaxants were used, as the surgeon did not request surgical muscle relaxation. The nasopharyngeal temperature of the child was monitored carefully and corrected if needed, and the anesthesia lasted for 25 min. Ophthalmologists used oxytetracycline hydrochloride eye drops during surgery. At the end of surgery, the patient underwent arterial blood gas analysis, and the results were normal, with the exception of a partial pressure of carbon dioxide value of 31 mmHg. The respiratory parameters were adjusted, and the anesthesia course and the postoperative period were uneventful. After 25 min of the postoperative period, the patient's breathing was stable, her oxygenation was satisfactory, and her laryngeal mask was removed. The child was then transferred to the post‐anesthesia care unit for further observation. The patient exited the operating theater in a good medical state, with vital signs within normal limits and full consciousness. Her blood homocysteine level was similar to the preoperative level, and her ammonia level decreased normally on the first postoperative day. She was subsequently discharged home and returned to the hospital every 6 months. Notably, no new changes in her condition have occurred (Figure 3).

## 3 DISCUSSION

MMA combined with hyperhomocysteinemia is caused by abnormal congenital metabolism of intracellular vitamin B12. These metabolic diseases are caused by mutations in the *MMACHC* gene, located on chromosome 1p34.1. In patients with this condition, vitamin B12 cannot be converted into deoxyadenosyl cobalamin, and the mitochondrial activity of methylmalonyl coenzyme A mutase decreases, which results in a large accumulation of methylmalonic acid and MMA. Furthermore, vitamin B_12_ cannot be converted into methylcobalamin, which decreases the activity of methionine synthetase and causes homocysteine accumulation in the body. MMA combined with hyperhomocysteinemia causes multisystem damage, and the symptoms mainly affect the eyes and brain. According to early or late symptom onset, the condition can be divided into early or late onset, respectively, and the early onset condition usually presents as severe and multiple organ damage symptoms in the first year after birth. Patients with late‐onset often exhibit symptoms between the ages of 4–14 years, sometimes even in adulthood. The late‐onset condition often first presents as nervous system symptoms, cognitive disorders, psychobehavioral abnormalities, and cerebellar damage.^6^ Oral betaine and L‐carnitine are the mainstay treatments for MMA combined with hyperhomocysteinemia. Currently, there is no evidence to recommend a specific anesthetic management for MMA combined with hyperhomocysteinemia; thus, it is imperative to understand the disease in advance and ensure the safe use of anesthetics.

The primary symptom of MMA is nervous system injury. Brain imaging examinations are crucial in assessing the extent of brain damage in children; therefore, it is essential to conduct preoperative brain imaging examinations. Unfortunately, due to a lack of knowledge about the disease, brain imaging examinations are not conducted typically conducted prior to operation. The electroencephalogram (EEG) of this child showed abnormalities. Despite the absence of epilepsy symptoms, the child had a family history of epilepsy. Therefore, vigilant monitoring of anesthesia depth during the operation is of utmost importance, and the anesthesia depth monitor can display the original EEG waveform. The presence of burst suppression on the monitor suggests when the anesthesia depth may be excessively deep or when the patient may be in an abnormal state, such as hypothermia, ischemia, or hypoxia. Furthermore, inadequate anesthesia depth can result in intraoperative awareness and possibly induce epileptic seizures. Therefore, it is crucial to maintain an appropriate anesthesia depth during the operation and remain cautious of both excessive and insufficient anesthesia. However, due to the short operation time and the need to attach anesthesia depth monitoring electrodes to the forehead, which compromises the range of surgical disinfection, the patient was not monitored for anesthesia depth. Sahutoglu et al. discovered that pediatric patients may experience hypothermia even after a brief procedure. Intraoperative hypothermia can cause increased blood lactate levels, resulting in metabolic acidosis.^7^ Consequently, monitoring temperature and depth of anesthesia are vital. To safeguard the child from hypothermia, we employ methods such as fluid heating, electric blankets, and adjustments to the temperature and humidity in the operating room.

Severe ketoacidosis in children with MMA is frequently accompanied by intracellular dehydration, underscoring the importance of volume management. Nevertheless, hypervolemia or metabolic alkalosis can lead to the development or worsening of cerebral edema. Consequently, it is recommended to implement goal‐directed fluid therapy and correct the acid–base balance.^8^ It is important to avoid prolonged fasting before surgery, as extended periods of fasting can lead to hyperglycemia, protein breakdown, gluconeogenesis, elevated blood ammonia levels, and worsened condition. Age‐appropriate intravenous glucose infusions can be administered prior to the operation, at various concentrations. In the context of maintaining normal blood sugar levels, insulin can be employed to stimulate anabolism. Consistently maintaining a stable, normal blood sugar level indirectly indicates effective anabolism. During the operation, the child received an intravenous infusion of 120 mL of 5% glucose and sodium chloride, which resulted in a rise in blood glucose levels from 4.5 mmol/L before the operation to 6.8 mmol/L after the operation.

During the operation, it is essential to monitor blood gas levels promptly to correct acidosis and maintain a stable internal environment. The alkalization of urine using bicarbonate can accelerate the elimination of methylmalonic acid in urine. However, excessive intravenous infusion of bicarbonate can lead to hypernatremia, cerebral edema, and even cerebral hemorrhage. Therefore, it is crucial to monitor blood gas levels in a timely manner and calculate the appropriate bicarbonate dosage based on the results. Additionally, close attention should be given to changes in lactic acid levels, and if lactic acid exceeds 5 mmol/L, insulin administration is recommended. Furthermore, continuous monitoring of urine output is necessary to ensure adequate perfusion of vital organs throughout the body. After the operation, it is important to remain vigilant about circulatory changes to promptly identify and address any issues. Nonsteroidal anti‐inflammatory drugs (NSAIDs), such as ibuprofen, acetaminophen, flurbiprofen axetil, and their derivatives, should be avoided in pediatric patients who require postoperative analgesia because their metabolites contain methylmalonic acid. For this patient, proparacaine hydrochloride eye drops were used during the operation, which resulted in a FLACC score of 1 postoperatively. Therefore, no analgesics were administered after the procedure. During surgery, factors that can induce or worsen pulmonary hypertension should be avoided, such as hypoxemia, hypercapnia, hypothermia, acidosis, high blood volume, insufficient blood volume, etc., to prevent the occurrence of pulmonary arterial hypertension crisis.

Betaine directly affects the concentration of homocysteine by promoting the formation of methionine from homocysteine, which weakens the stress response induced by homocysteine. At the same time, betaine converts homocysteine into methionine, which plays an important role in antioxidant activity. Combining the use of metabolic cofactors, such as vitamin B12 and levocarnitine, can maintain stable condition. In the present case, betaine, vitamin B12, and levocarnitine were applied throughout the perioperative period.

Patients with MMA have mitochondrial energy metabolism disorders, and medications that interfere with mitochondrial function can worsen the disease. Erythromycin, as a mitochondria‐targeted antibiotic, selectively binds to the large and small subunits of mitochondrial ribosomes, leading to mitochondrial dysfunction. Therefore, the use of erythromycin eye ointment should be approached with caution after surgery. Local anesthetics, when administered in excessive amounts or injected intravascularly, can disrupt mitochondrial energy metabolism mainly due to their toxicity to nerve cells, skeletal muscles, or cardiomyocytes.^9^ Hence, it is crucial to strictly control the dosage of local anesthetics and promptly identify local anesthetic drug poisoning. The use of propofol remains a topic of debate. It was reported that propofol was associated with metabolic decompensation in liver transplant patients with MMA.^10^ Some studies suggest that short‐term use of propofol is safe for MMA patients. Propofol can regulate mitochondrial fatty acid metabolism to protect myocardial function, as free fatty acids have toxic effects on the heart muscle.^9^ The safety of using propofol for anesthesia induction in stable patients was demonstrated. The occurrence of malignant high fever is closely related to inhaled general anesthetics, especially in patients with genetic abnormalities. Therefore, it is not recommended to use inhaled anesthetics in MMA patients to avoid the occurrence of malignant high fever during surgery.

## 4 CONCLUSIONS

To conclude, it is essential to thoroughly evaluate children with MMA prior to surgery, with particular attention to brain imaging examinations. In addition to routine monitoring during the procedure, temperature, anesthesia depth, and urine output should also be closely monitored as necessary. Total intravenous anesthesia is preferable, and caution should be exercised when using inhaled anesthetic drugs. Timely blood gas analysis should be conducted throughout the perioperative period to correct any acid–base imbalances and maintain internal environment stability. During anesthesia management, hypercapnia should be avoided, and low temperature, hypoxia, low blood volume, hypoglycemia, and other factors can easily lead to metabolic disorders and pulmonary hypertension.

## AUTHOR CONTRIBUTIONS

**Yunting Pang:** Writing – original draft. **Fanqing Meng:** Writing – original draft. **Yaqiu Guo:** Writing – review and editing. **Feng Zhou:** Writing – original draft; writing – review and editing.

## FUNDING INFORMATION

This study was supported by grants from Jinan Science and Technology Plan (202134071 to FM).

## CONFLICT OF INTEREST STATEMENT

All authors declared no conflict of interest related to this article.

## CONSENT

Written informed consent was obtained from the patient and her father for publication of this case report and any accompanying images.

## Data Availability

All data generated or analyzed during this study are included in this published article.
